# Postoperative high-sensitivity troponin T predicts 1-year mortality and days alive and out of hospital after orthotopic heart transplantation

**DOI:** 10.1186/s40001-022-00978-4

**Published:** 2023-01-09

**Authors:** René M’Pembele, Sebastian Roth, Anthony Nucaro, Alexandra Stroda, Theresa Tenge, Giovanna Lurati Buse, Florian Bönner, Daniel Scheiber, Christina Ballázs, Igor Tudorache, Hug Aubin, Artur Lichtenberg, Ragnar Huhn, Udo Boeken

**Affiliations:** 1 Department of Anesthesiology, Medical Faculty and University Hospital Duesseldorf, Heinrich-Heine-University Duesseldorf, Duesseldorf, Germany; 2 Department of Cardiology, Pulmonology and Vascular Medicine, Medical Faculty and University Hospital Duesseldorf, Heinrich-Heine-University Duesseldorf, Duesseldorf, Germany; 3 Department of Cardiac Surgery, Medical Faculty and University Hospital Duesseldorf, Heinrich-Heine-University Duesseldorf, Duesseldorf, Germany; 4 Department of Anesthesiology, Kerckhoff Heart and Lung Center, Bad Nauheim, Germany

**Keywords:** Heart failure, Heart transplantation, IMPACT score, Risk prediction, Patient-centered outcomes

## Abstract

**Background:**

Orthotopic heart transplantation (HTX) is the gold standard to treat end-stage heart failure. Numerous risk stratification tools have been developed in the past years. However, their clinical utility is limited by their poor discriminative ability. High sensitivity troponin T (hsTnT) is the most specific biomarker to detect myocardial cell injury. However, its prognostic relevance after HTX is not fully elucidated. Thus, this study evaluated the predictive value of postoperative hsTnT for 1-year survival and days alive and out of hospital (DAOH) after HTX.

**Methods:**

This retrospective cohort study included patients who underwent HTX at the University Hospital Duesseldorf, Germany between 2011 and 2021. The main exposure was hsTnT concentration at 48 h after HTX. The primary endpoints were mortality and DAOH within 1 year after surgery. Receiver operating characteristic (ROC) curve analysis, logistic regression model and linear regression with adjustment for risk index for mortality prediction after cardiac transplantation (IMPACT) were performed.

**Results:**

Out of 231 patients screened, 212 were included into analysis (mean age 55 ± 11 years, 73% male). One-year mortality was 19.7% (40 patients) and median DAOH was 298 days (229–322). ROC analysis revealed strongest discrimination for mortality by hsTnT at 48 h after HTX [AUC = 0.79 95% CI 0.71–0.87]. According to Youden Index, the cutoff for hsTnT at 48 h and mortality was 1640 ng/l. After adjustment for IMPACT score multivariate logistic and linear regression showed independent associations between hsTnT and mortality/DAOH with odds ratio of 8.10 [95%CI 2.99–21.89] and unstandardized regression coefficient of −1.54 [95%CI −2.02 to −1.06], respectively.

**Conclusion:**

Postoperative hsTnT might be suitable as an early prognostic marker after HTX and is independently associated with 1-year mortality and poor DAOH.

**Supplementary Information:**

The online version contains supplementary material available at 10.1186/s40001-022-00978-4.

## Introduction

Orthotopic heart transplantation (HTX) is still the gold standard therapy for end-stage heart failure. Unfortunately, there is an ongoing donor shortage which limits the number of HTX and leads to an increasing number of patients on HTX waiting lists [1]. In addition, the treatment of end-stage heart failure is continuously improving so that the patients undergoing HTX are getting older and have more comorbidities with an increased risk for postoperative complications [2, 3]. To optimize perioperative risk stratification and early re-estimation of risk, the development of risk prediction models and the identification of perioperative prognostic factors became more and more important [^4^, ^5^, ^6^]. Numerous risk stratification tools have been developed in the past years, but their clinical use is often limited by insufficient predictive values [5]. In this context, the Index for Mortality Prediction After Cardiac Transplantation (IMPACT) score was introduced as validated tool for prediction of 1-year mortality after HTX [7, 8]. However, some studies report poor-to-moderate discrimination for mortality in their cohorts [9–11].

Previously biomarkers were established as another possibility to support perioperative stratification and early re-estimation of risk. A sensitive biomarker for myocardial cell injury is high-sensitivity troponin T (hsTnT) [12]. Postoperative troponin release has been investigated extensively in cardiac and non-cardiac surgery and is associated with adverse events [^13^, ^14^, ^15^]. Recently, Devereaux et al. investigated the prognostic value of high-sensitivity troponin I (hsTnI) in patients undergoing cardiac surgery and showed that levels of hsTnI were independently associated with mortality [16]. The role of hsTnT as a prognostic factor after HTX, however, is not clear and recent literature is ambiguous [17]. Therefore, we conducted this analysis to evaluate whether hsTnT is a suitable marker for risk stratification and prognosis after HTX.

## Methods

This retrospective single-center cohort study was approved by the University of Duesseldorf’s ethics committee (reference number: 4567) and complies with the International Society for Heart and Lung Transplantation (ISHLT) ethics statement. Data were extracted from the local prospective HTX database. All patients had given written informed consent to be registered in this database. Reporting of this work corresponds to the “Strengthening the Reporting of Observational Studies in Epidemiology” (STROBE) guidelines [18].

### Patient population

All patients aged  ≥ 18 years who underwent HTX at the University Hospital Duesseldorf, Germany, in a time period from September 2010 to August 2021 were considered for inclusion. Patients with missing data regarding survival, DAOH and hsTnT measurements, as well as patients without completed 1-year follow-up were excluded from analysis. In all patients HTX was conducted using bicaval technique and traditional cold storage was used for donor organ preservation.

### High-sensitivity troponin T measurements

Main exposure was postoperative hsTnT measured in ng/L after HTX at different time points. At our institution hsTnT is routinely measured preoperatively, within the first 12 h, day 1, day 2 and day 3 after HTX. Measurements were performed in the central laboratory of the University hospital Duesseldorf.

### IMPACT score calculation

The risk index for mortality prediction after cardiac transplantation (IMPACT) is a score validated to predict 1-year mortality after HTX from preoperative recipient risk factors. This score assigns varying points for 10 variables: age, serum bilirubin, creatinine clearance, dialysis, sex, heart failure etiology, preoperative infection, race, circulatory support and type of ventricular assist device. The score was calculated for each HTX patient as described before with a maximum of 50 points [7, 8].

### Outcomes

The primary outcome of this study was mortality during the first year after HTX. Days alive and out of hospital (DAOH) at 1 year after HTX was the secondary endpoint of this study. Calculation of DAOH was conducted by summing up all days of hospitalization in the first year after HTX and subtracting them from 365 days, as described before [19–21]. In case of mortality, the number of days the patient did not survive and of days spent in hospital were subtracted from 365 days.

### Statistical analysis

Statistical analysis was performed using IBM SPSS^©^ software version 25.0 (Armonk, NY, USA), GraphPad Prism^©^ version 8.02 (La Jolla, California, USA), MedCalc^®^ Statistical Software version 20.114 (MedCalc Software Ltd, Ostend, Belgium) and R Statistical Software (v4.1.2; R Core Team 2021). Patients characteristics with continuous variables were presented as mean ± standard deviation (SD) or as median and interquartile ranges (IQR, 25–75%), as appropriate. Categorical variables were presented as numbers (*n*) with corresponding percentages (%) in brackets. Fisher’s exact test or unpaired t-tests were used to test for differences between dichotomous or continuous variables between groups defined by survival status. For analysis of the primary endpoint, receiver operating characteristic (ROC) analyses were performed for hsTnT levels within 12 h, 24 h, 48 h and 72 h after HTX. Cutoff values for troponin levels were determined by Youden index. The cutoff of the hsTnT time point with the strongest discrimination for 1-year mortality in ROC analysis was added to a logistic regression model, with adjustment using the continuous IMPACT score. The net reclassification improvement (NRI) and the net absolute reclassification improvement (NARI) of the mortality prediction model by adding the postoperative troponin cutoff were assessed. Discrimination (ROC-AUC) of the models with and without postoperative hsTnT was quantified and compared using Delong test. To compare net benefit of using these models to detect patients’ risk for 1-year mortality, a decision curve analysis was performed for both models.

For analysis of DAOH patients were classified by hsTnT cutoff. DAOH was compared using non-parametric Mann–Whitney U test. Association of continuous hsTnT elevation per 100 ng/L with DAOH was adjusted by the continuous IMPACT score using multivariable linear regression. For all statistical tests, a *p* < 0.05 was considered significant.

## Results

### Study cohort and characteristics

In total 231 patients underwent HTX at the University Hospital Duesseldorf during the time period of September 2010 and December 2021. Thereof, 19 (8%) patients had to be excluded according to the exclusion criteria. Among the 212 included patients, mean age was 55 ± 11 years and 155 (73%) were male. During first year after HTX, 40 patients (19%) died. Causes of death were: graft dysfunction (7 patients), septic shock (9 patients), major bleeding complications (6 patients with intracranial hemorrhage, 1 patient with gastrointestinal bleeding, 1 patient with ECMO cannulation site bleeding), bowel ischemia (3 patients), cerebral hypoxia (3 patients) and unknown causes (10 patients who died out of hospital). Overall median DAOH was 298 days (229–322). Detailed patient characteristics for survivors and non-survivors are presented in Table 1 (Additional file 1: Figure S1, Table 1).

**Table 1:** Characteristics of survivors and non-survivors after HTX

	Survivors (*N* = 172)	Non-survivors (*N* = 40)	*p*-value^a^
Preoperative characteristics			
Male sex	128 (74)	27 (68)	0.429
Age (years)	55 ± 11	57 ± 11	0.140
BMI (kg/m^2^)	25.6 ± 4.5	25.8 ± 5.1	0.861
Smoker	41 (24)	8 (20)	0.681
Diabetes	34 (20)	14 (35)	0.058
Arterial hypertension	106 (62)	21 (53)	0.286
Pulmonary hypertension	14 (8)	5 (13)	0.368
Donor–recipient sex mismatch	45 (26)	14 (35)	0.327
Prior cardiothoracic surgery	108 (63)	29 (73)	0.276
LVAD	86 (50)	25 (63)	0.164
Preoperative ECMO	5 (3)	3 (8)	0.175
Preoperative mechanical ventilation	7 (4)	3 (8)	0.405
Preoperative dialysis	7 (4)	4 (10)	0.127
Serum creatinine (mg/dl)	1.4 ± 1.0	1.3 ± 0.5	0.649
Creatinine clearance (ml/min)	65 ± 24	64 ± 31	0.793
Bilirubin (mg/dl)	0.8 ± 0.9	1.2 ± 1.2	0.080
IMPACT score	8 ± 5	10 ± 5	0.003
Donor characteristics			
Male sex	99 (58)	19 (48)	0.290
Age (years)	41 ± 12	50 ± 10	< 0.0001
BMI (kg/m^2^)	26 ± 5	25 ± 4	0.365
Smoker	93 (54)	17 (43)	0.220
Arterial hypertension	40 (23)	16 (40)	0.045
Diabetes	11 (6)	3 (8)	0.731
Cardiopulmonary resuscitation	55 (32)	8 (20)	0.179
Intraoperative characteristics (min)			
Duration of surgery	424 ± 105	484 ± 144	0.017
Duration of CPB	250 ± 64	301 ± 99	0.003
Total ischemia time	212 ± 49	228 ± 52	0.070
Reperfusion time	126 ± 45	151 ± 69	0.044
Postoperative characteristics			
ECMO	38 (22)	23 (58)	< 0.0001
Renal replacement therapy	87 (53)	31 (80)	0.003
Neurological complication	20 (12)	17 (43)	< 0.0001
Days in ICU	23 ± 22	30 ± 38	0.116
Duration of mechanical ventilation (hours)	119 ± 168	268 ± 236	0.001

*BMI* body mass index, *CPB* cardiopulmonary bypass, *ECMO* extracorporeal membrane oxygenation, *IMPACT* risk index for mortality prediction after cardiac transplantation, *ICU* intensive care unit, *LVAD* left ventricular assist device

### HsTnT levels of survivors and non-survivors after HTX

**Fig. 1:**
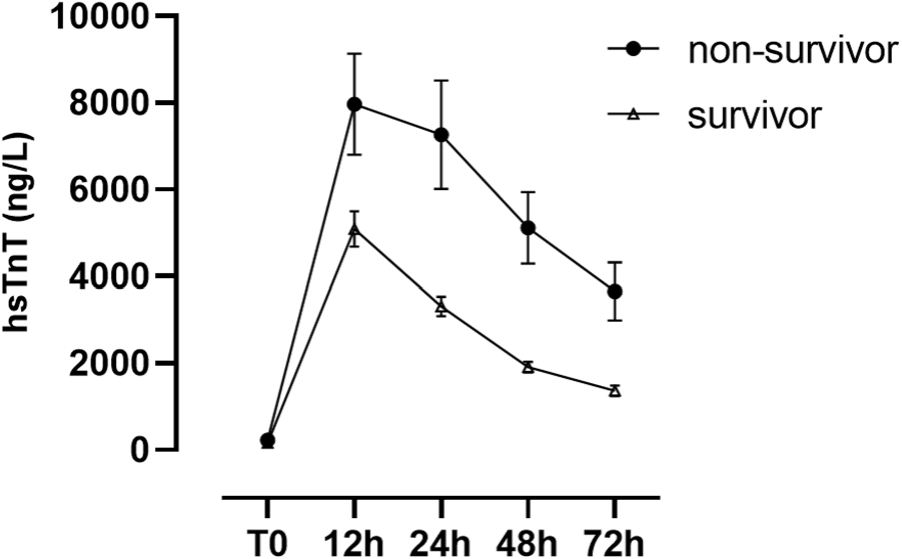
Postoperative levels of hsTnT of survivors and non-survivors. The graphs depict high-sensitivity troponin (hs-TnT) concentrations for survivors (triangles) and non-survivors (dots) across different time points with corresponding standard errors. Preoperative hs-TnT values did not differ significantly between groups [baseline hsTnT—survivors 165 ± 687 ng/L vs. non-survivors 228 ± 906 ng/L, *p* = 0.638]. Postoperative values at timepoints 12 h, 24 h, 48 h and 72 h after surgery were significantly higher in non-survivors as compared to survivors [hsTnT 12 h—survivors 5089 ± 5305 ng/L vs. non-survivors 7972 ± 7419 ng/L, *p* = 0.005; hsTnT 24 h—survivors 3309 ± 2934 ng/L vs. non-survivors 7266 ± 7942 ng/L, *p* ≤ 0.0001; hsTnT 48 h—survivors 1911 ± 1598 ng/L vs. non-survivors 5115 ± 5196 ng/L, *p* ≤ 0.0001; hsTnT 72 h—survivors 1363 ± 1565 ng/L vs. non-survivors 3651 ± 4126 ng/L, *p* ≤ 0.0001]

Baseline levels of hsTnT did not differ significantly between survivors and non-survivors. In contrast, postoperative hsTnT levels were significantly higher in patients who died as compared to survivors at 12 h, 24 h, 48 h and 72 h after HTX. Detailed results are presented in Fig. 1.

### ROC analysis for postoperative hsTnT and 1-year mortality

**Fig. 2:**
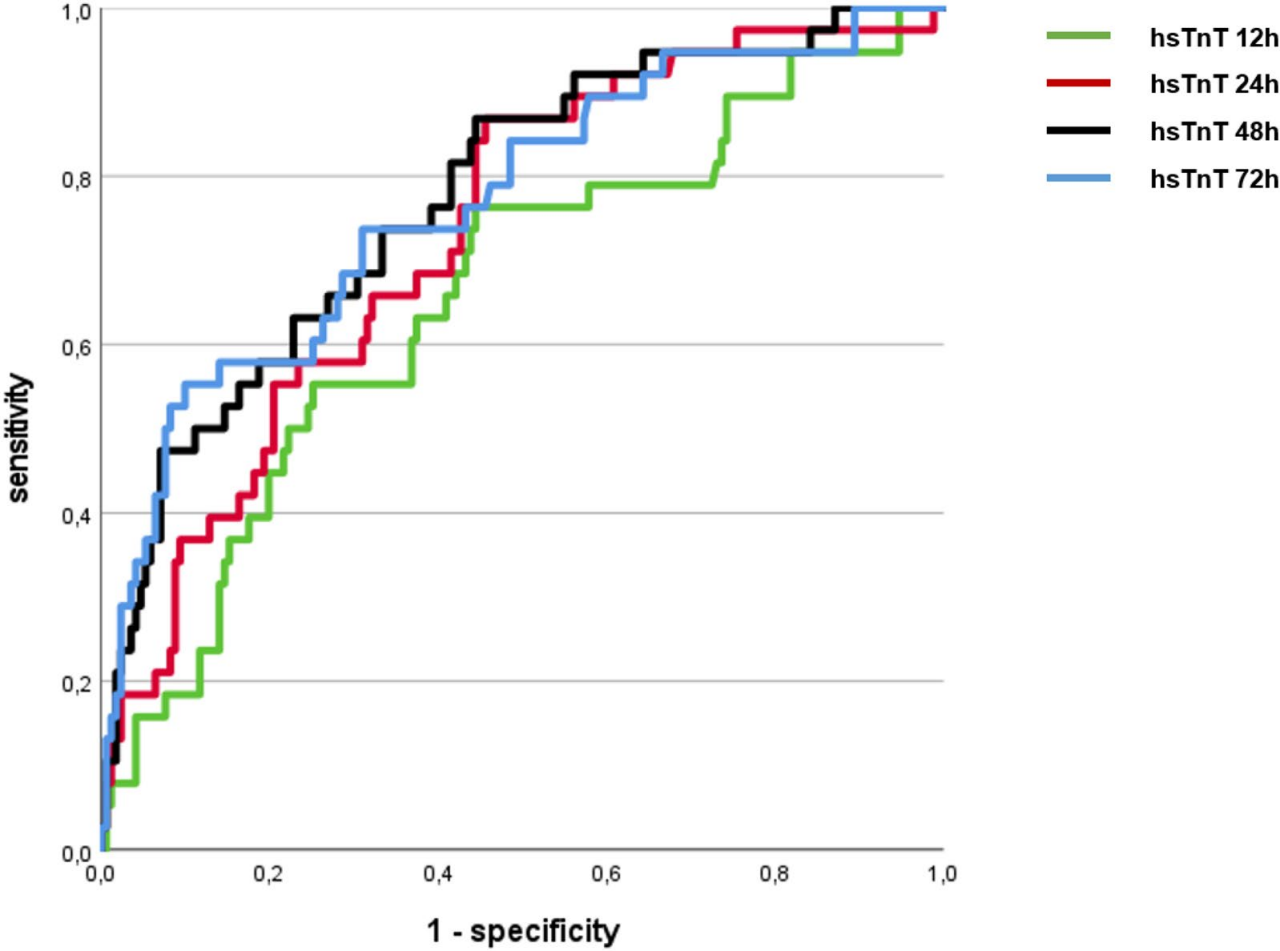
Receiver operating characteristic curves of postoperative hsTnT and 1-year mortality. The figure shows the receiver operating characteristics (ROC) curves for association of different hs-TnT sampling time points with 1-year mortality after heart transplantation. The areas under the curves (AUC) are as follows: hsTnT 12 h AUC = 0.66 (CI 0.56–0.75); hsTnT 24 h AUC = 0.74 (CI 0.66–0.82); hsTnT 48 h AUC = 0.79 (CI 0.71–0.87); hsTnT 72 h AUC = 0.77 (CI 0.68–0.86). The numerically strongest discrimination ability is given for hsTnT values at 48 h after heart transplantation

We performed ROC analysis for hsTnT levels and 1-year mortality at sampling time points 12 h, 24 h, 48 h and 72 h after HTX, respectively. All postoperative hsTnT showed a significant discrimination for 1-year mortality [hsTnT_12h_—AUC = 0.66, 95% CI 0.56–0.75; hsTnT_24h_—AUC = 0.74, 95% CI 0.66–0.82; hsTnT_48h_—AUC = 0.79, 95% CI 0.71–0.87; hsTnT_72h_—AUC = 0.77, 95% CI 0.68–0.86]. The hsTnT levels at 48 h after HTX showed numerically strongest discrimination for 1-year mortality regarding the AUC. Youden Index determined a cutoff of 1640 ng/L for hsTnT at 48 h after HTX (Fig. 2).

### Binary logistic regression model for hsTnT and 1-year mortality

**Fig. 3:**
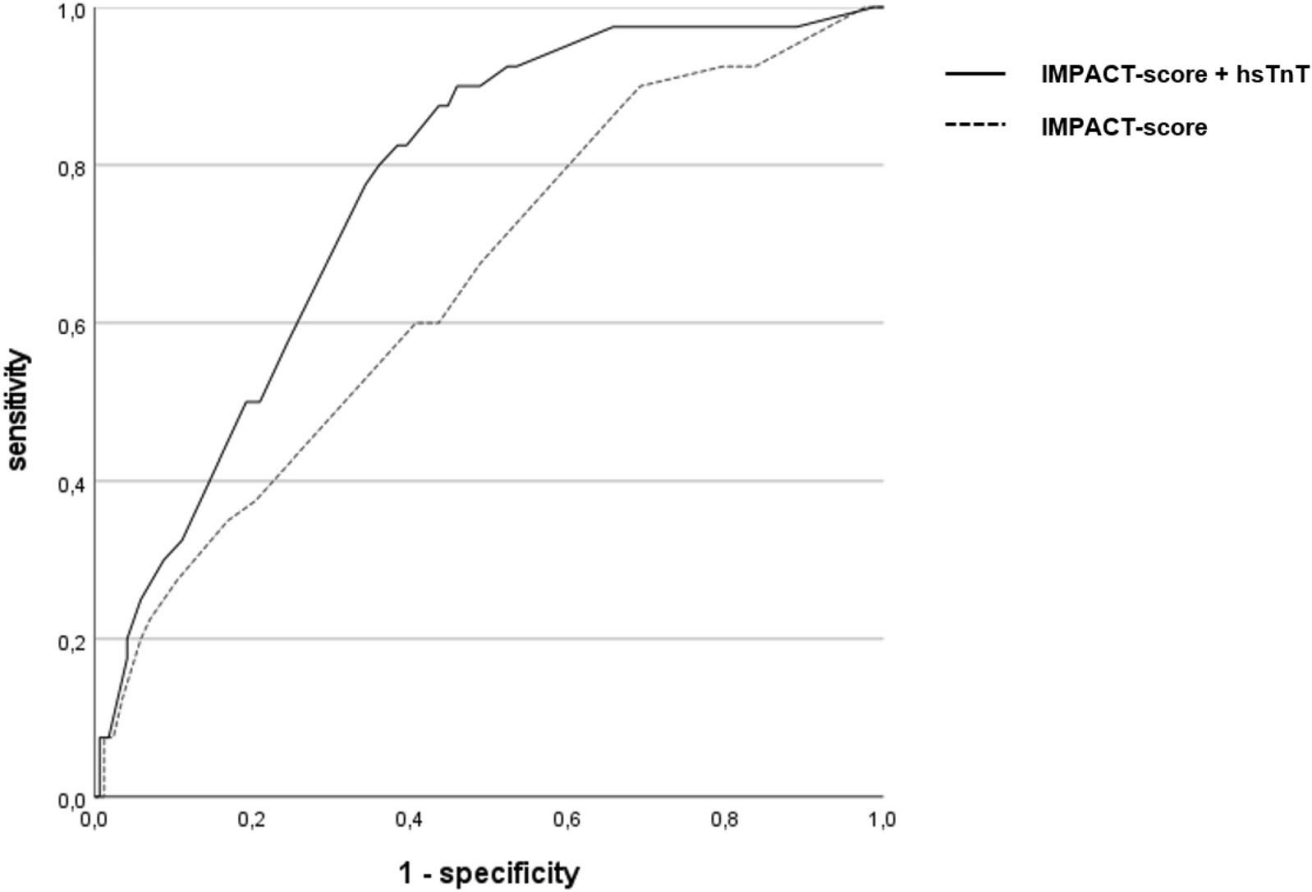
Receiver operating characteristic curves of two prediction models for 1-year mortality. The figure shows the receiver operating characteristics (ROC) curves of risk prediction models for 1-year mortality. While on its own the IMPACT score has a ROC- a moderate discrimination ability (AUC = 0.65 95% CI 0.56–0.74), adding hsTnT levels at 48 h after heart transplantation to the model improves its performance (AUC = 0.79; CI 0.71–0.87)

**Fig. 4:**
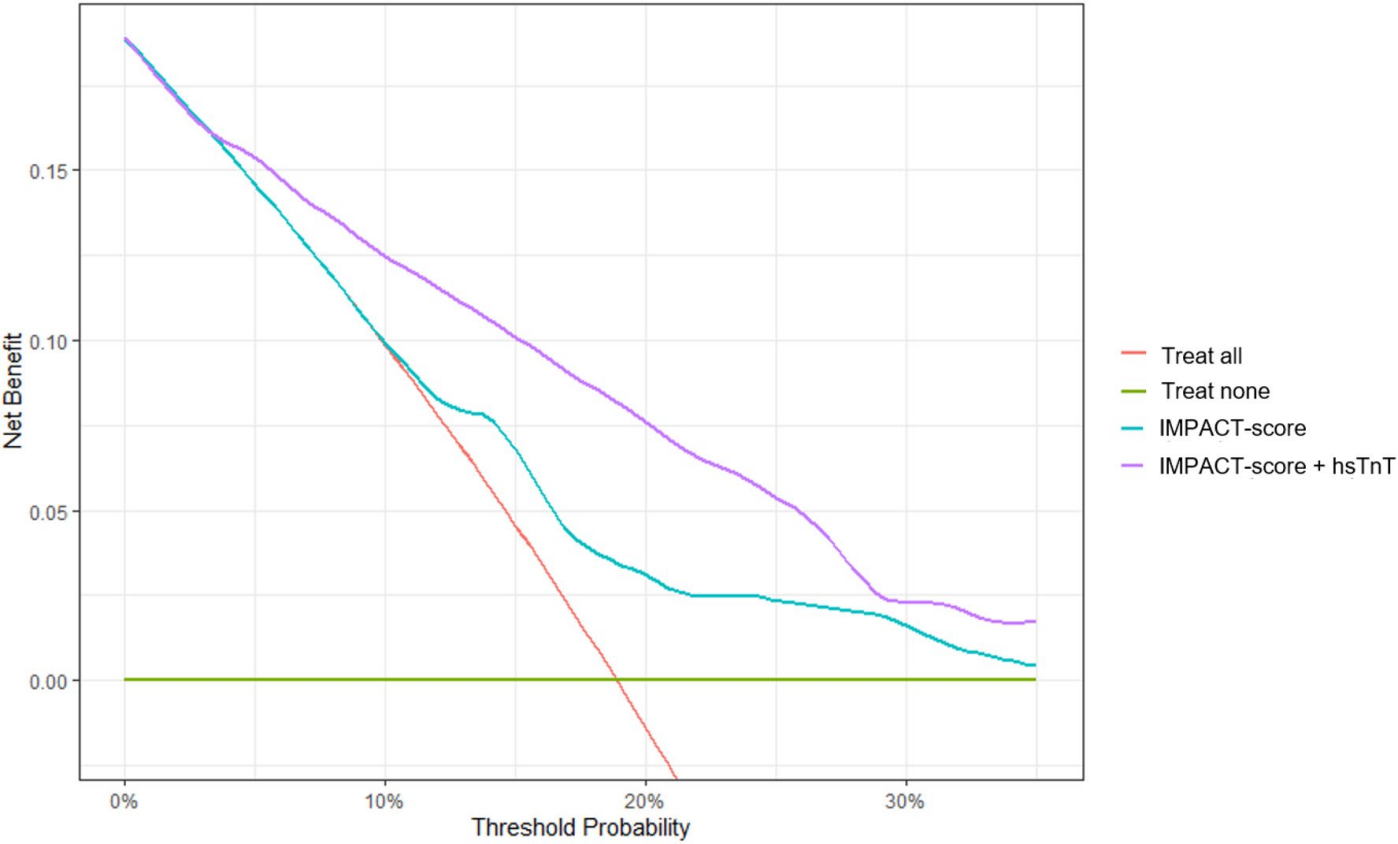
Decision curves for different models of risk prediction after HTX. The figure shows a decision curve analysis for mortality prediction model with IMPACT score (turquoise) and IMPACT score with hsTnT (purple). The x-axis shows the threshold probability for 1-year mortality while the y-axis shows the net benefit of the models. Beyond a threshold of 5% the combined model of IMPACT and hsTnT shows the greatest net benefit. The red line depicts a model in which all patients would be treated and the green line represents a model in which none patient will be treated

Binary logistic regression model was performed with hsTnT cutoff at 48 h after HTX as independent variable and 1-year mortality as dependent variable. In univariate analysis hsTnT levels at 48 h after HTX showed significant association with 1-year mortality [OR 8.84 95% CI 3.31–23.66, *p* ≤ 0.0001]. After adjustment for continuous IMPACT score, association of hsTnT with mortality remained significant [hsTnT_48h_—OR 8.10 95% CI 2.99–21.89, *p* ≤ 0.0001; IMPACT score—OR 1.09 95% CI 1.01–1.18, *p* = 0.025]. We analyzed in how far risk prediction for 1-year mortality by IMPACT score was improved by the addition of hsTnT levels at 48 h after HTX are added to the logistic regression model. The NRI for the model including hsTnT was 7.6% (95% CI 4.1–12.6) for non-events and 27.5% (95% CI 14.6–43.9) for events. Regarding NARI, the model was able to identify 114/1000 patients more at risk for 1-year mortality. Corresponding reclassification tables were added to the supplements. In ROC analysis the AUC for the model including troponin was significantly higher as compared to the model only including IMPACT score [IMPACT score—AUC = 0.65 95% CI 0.56–0.74; IMPACT score with hsTnT_48h_—AUC = 0.77 95% CI 0.70–0.84; difference between areas: 0.12, 95% CI 0.04–0.19, *p* = 0.0016]. The net benefit curve suggested highest net benefit for the combined use of IMPACT and hsTnT (Figs. 3, 4, Additional file 1: Table S1).

### Association of postoperative hsTnT and DAOH

**Fig. 5:**
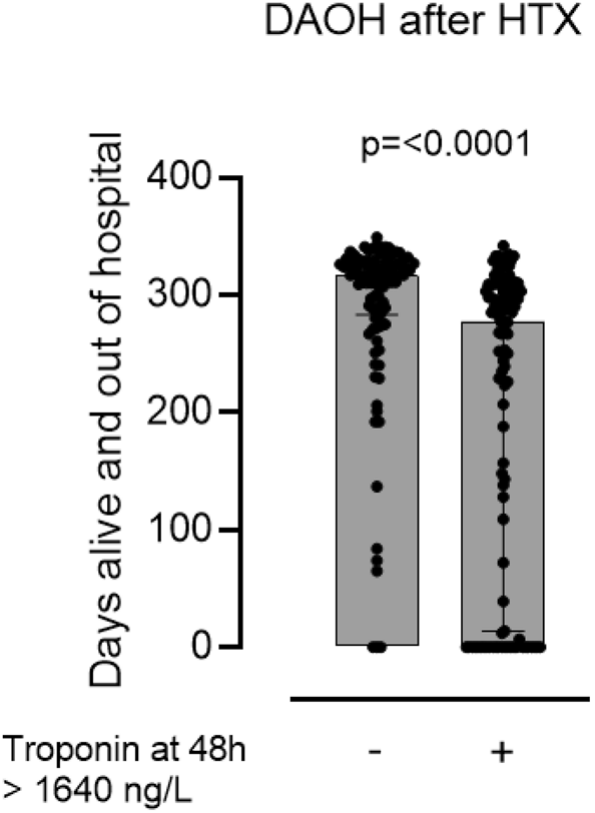
Association of hsTnT levels above cutoff and days alive and out of hospital. The box-plot shows significantly fewer DAOH for patients above the determined cutoff of hs-TnT at 48 h after heart transplantation [hsTnT 48 h—below cutoff 317 (283–328) days vs. above cutoff 278 (14–308) days, *p* ≤ 0.0001]. Black dots represent individual DAOH values of patients, the upper end of the boxes shows the median while error bars depict interquartile ranges. The hsTnT cutoff was determined by Youden index

In univariate analysis hsTnT levels higher than predefined cutoff by Youden Index were associated with lower DAOH at 48 h after HTX [hsTnT_48h_—below cutoff 317 (283–328) days vs. above cutoff 278 (14–308) days, *p* ≤ 0.0001]. Results for other hsTnT sampling timepoints are presented in the supplements. In a multivariable linear regression model, association of continuous hsTnT elevation (per 100 ng/L) and DAOH remained significant when adjusted for points on the IMPACT score [per 100n g/L hsTnT elevation—regression coefficient: -1.54, 95% CI −2.02 to −1.06, *p* ≤ 0.0001; IMPACT score—regression coefficient: −4.79, 95% CI −7.83 to −1.76, *p* = 0.002] (Fig. 5, Additional file 1: Figure S2, Table 2).

**Table 2:** Multivariable linear regression model for influence of postoperative troponin on days alive and out of hospital

Variables for multivariable linear regression	Unstandardized coefficients B	Standard error	Standardized coefficients beta	95% CI	*p*-value
Per 100 ng/L hsTnT elevation	−1.54	0.25	−0.39	−2.02 to −1.06	< 0.0001
IMPACT score	−4.79	1.54	−0.19	−7.83 to −1.76	0.002

*hsTnT* high-sensitivity troponin T, *IMPACT* risk index for mortality prediction after cardiac transplantation

## Discussion

The present study suggested that early hsTnT levels at 48 h after HTX are independently associated with mortality and DAOH after HTX. Moreover, this study showed that the addition of hsTnT improved risk prediction for mortality and DAOH over IMPACT score.

Referring to the current literature, the prognostic value of troponin after HTX is underexplored. A recent systematic review by Liu and colleagues identified only three studies including a total of 372 patients that investigated the association between elevated troponin levels and mortality. As these studies revealed significant heterogeneities, the authors decided not to perform a meta-analysis [17]. Labarrere et al. investigated the value of persistent troponin I (TnI) levels in 110 HTX patients during first year after HTX. They found that persistent TnI levels greater than 0.5 ng/ml were associated with development of coronary artery disease and graft failure. However, as patients were only included if they had survived the first year after HTX, association of postoperatively elevated TnI and early mortality was not investigated [22]. Another study investigated the prognostic value of postoperative hsTnT for 1-year mortality in 141 HTX patients. They identified that elevated hsTnT levels at 6 weeks after HTX were highly associated with 1-year mortality. Again, association of early postoperative levels of hsTnT was not investigated by the authors [23]. The last study by Franeková et al. demonstrated an association of hsTnT levels at 10 days after HTX and 1-year mortality [24]. However, an earlier postoperative assessment of risk might be favorable as it might change clinical practice for patients at risk.

Postoperative troponin release has been extensively studied in non-cardiac surgery before [^25^, ^26^]. In these studies, early postoperative troponin elevation above the upper limit of normal was associated with major adverse events like mortality. In cardiac surgery however, this upper limit of normal troponin concentration is frequently exceeded by myocardial trauma due to surgery, with not necessarily higher risk for mortality [13]. Therefore, Devereaux et al. defined new cutoffs for association of troponin and 30-day mortality after cardiac surgery corresponding with an hsTnI level 218 times the upper reference limit [16]. Our recent study now adds data for association of early hsTnT with 1-year mortality after HTX. Patients who died within the first year after HTX had significantly higher troponin values at each timepoint of measurement. These findings were independently associated when adjusted for the IMPACT score. IMPACT score showed weak-to-moderate association with 1-year mortality in our cohort. This goes in line with previous reports, describing similar AUC in ROC analysis [9, 11]. Addition of hsTnT level at 48 h improved the discrimination ability of IMPACT score. The net benefit using the combined model was also higher to identify patients at risk for 1-year mortality. Recently, similar risk prediction models and decision curve analyses for mortality were presented as effective to guide palliative care consultation [27, 28]. Additionally, hsTnT levels were independently associated with low DAOH. This is an important finding, as this complements the current knowledge on more patient-centered outcomes in the field of end-stage heart failure and HTX surgery [21, 29, 30].

In the Eurotransplant area, the responsible parties discuss the implementation of a cardiac allocation score (CAS) which is supposed to optimize the allocation of the limited donor organs. In the lung transplantation setting, a similar score already exists (Lung allocation score (LAS)) which is also used to prioritize waiting list candidates 12 years and older based on a combination of waitlist urgency and post-transplant survival. Based on the data of this study, postoperative hsTnT may also be included into such a score for early re-estimation of postoperative risk and prognosis. Further studies should focus on potential interventions depending on hsTnT values that might be able to prevent complications and finally to reduce mortality after HTX. These may include intensified monitoring or standardized protocols for strict follow-up of these patients.

### Strengths and limitations

Strengths of the presented data include first, standardized troponin measurement at multiple time points with high data completeness; second, complete 12-month follow-up. Further, we did not only address mortality but also DOAH, a more patient-centered endpoint to quantify life impact [19]. We are aware of the following limitations. First, as a single-center study, sample size and number of events was limited. However, only 2 variables (troponin and IMPACT) were included into the logistic model that can therefore be considered robust. Second, we cannot exclude that any external hospitalization took place. However, HTX patients are very closely connected to our center so that we consider the risk of misclassification bias was very limited. Further, although DAOH is a measure of life impact, we did not collect data on quality of life, another relevant patient-centered outcome. Finally, we chose hsTnT at 48 h after HTX as primary biomarker for our analysis as it showed the numerically strongest discrimination for 1-year mortality. However, ROC-AUC did not significantly differ from values at 24 h and 72 h. In this context an earlier timepoint like 24 h after HTX might be favorable for early re-estimation of postoperative risk in the clinical setting. Therefore, optimal cutoff and sampling time point should be investigated in a larger cohort.

The generalizability of these findings may be hampered by the single-center design. However, characteristics such as 1-year mortality were in line with the current literature.

## Conclusion

Early hsTnT levels after HTX surgery are independently associated with poor 1-year survival and reduced DAOH. Therefore, early hsTnT concentrations might be useful for early risk reassessment to tailor postoperative therapy or decision-making in the intensive care unit.

## Supplementary Information

**Additional file 1: Figure S1.** Study Flowchart. **Figure S2. **Association of hsTnT levels at different timepoints above cutoff and days alive and out of hospital. **Table S1.** Reclassification tables of a 1-year mortality prediction model using IMPACT compared to a model using IMPACT and hsTnT.

## Data Availability

All relevant data are included in the present manuscript or in the supplements. Raw data are available upon reasonable request by the first author R.M.
