# Supplementary material for: Postoperative high-sensitivity troponin T predicts 1-year mortality and days alive and out of hospital after orthotopic heart transplantation
Source: Eur J Med Res. 2023 Jan 9;28:16. doi: 10.1186/s40001-022-00978-4 (PMC9827673; doi:10.1186/s40001-022-00978-4)
Supplement: Supplementary file 1 — Additional file 1: Figure S1. Study Flowchart. Figure S2. Association of hsTnT levels at different timepoints above cutoff and days alive and out of hospital. Table S1. Reclassification tables of a 1-year mortality prediction model using IMPACT compared to a model using IMPACT and hsTnT. [file 40001_2022_978_MOESM1_ESM.docx]

**Additional file:**

**Figure S1: Study Flowchart**


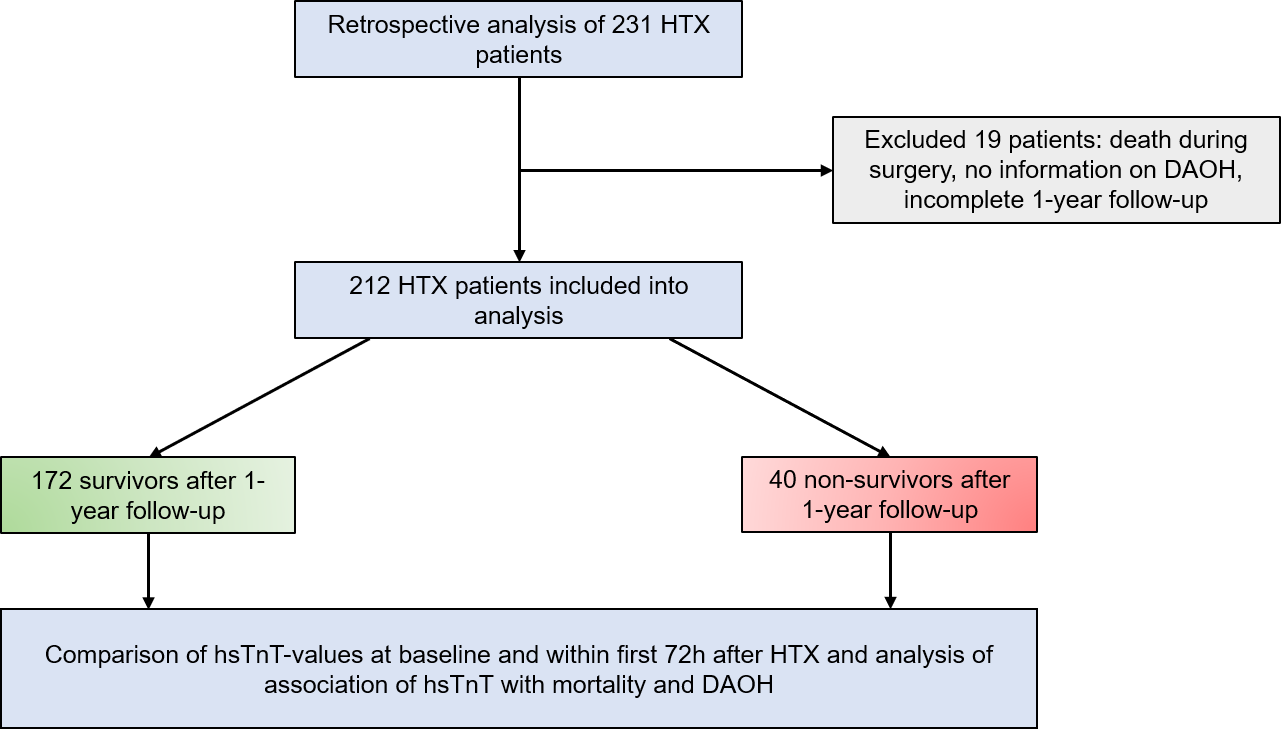


**Legend: study flowchart**

**Figure S2: Association of hsTnT levels at different timepoints above cutoff and days alive and out of hospital.**

**
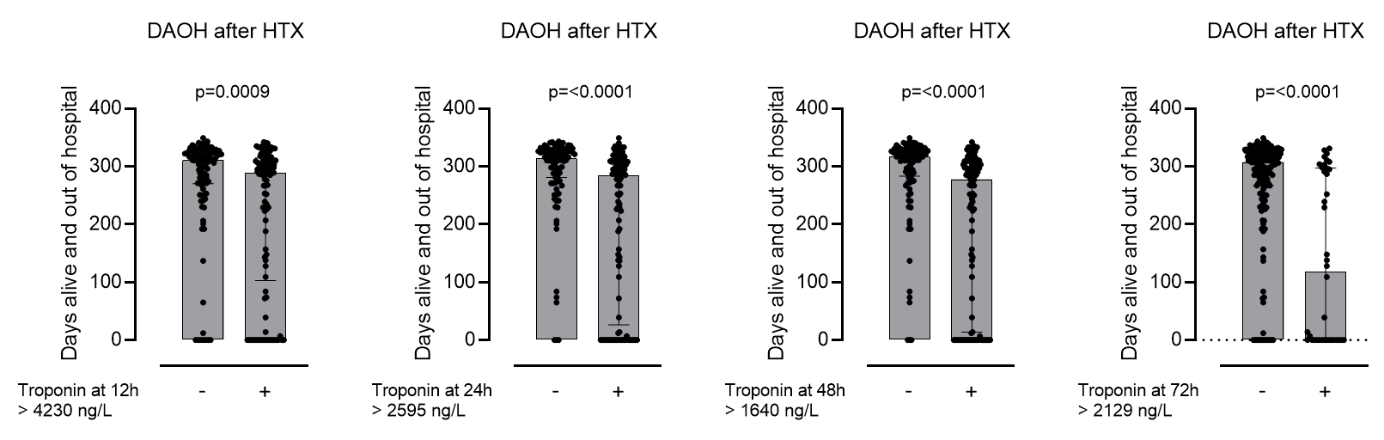
**

**Legend:** The box-plot shows significantly fewer DAOH for patients above the determined cut-offs of hs-TnT at different timepoints after heart transplantation [hsTnT 12h – below cutoff 311 (270-326) days vs. above cutoff 289 (103-315) days, p=0.0009; hsTnT 24h – below cutoff 314 (281-327) days vs. above cutoff 285 (27-310) days, p=<0.0001; hsTnT 48h – below cutoff 317 (283-328) days vs. above cutoff 278 (14-308) days, p=<0.0001; hsTnT 72h – below cutoff 308 (267-324) days vs. above cutoff 119 (0-297) days, p=<0.0001]. Black dots represent individual DAOH values of patients, the upper end of the boxes shows the median while error bars depict interquartile ranges. The hsTnT cutoff was determined by Youden-index.

**Table S1: Reclassification tables of a 1-year mortality prediction model using IMPACT compared to a model using IMPACT and hsTnT.**

**A**

| **Reclassification table non-events** | | | | | |
| --- | --- | --- | --- | --- | --- |
| **Model** |  | IMPACT + hsTnT | | | Total patients |
|  | **Risk group** | low | intermediate | high |  |
| IMPACT | Low | 39 | 18 | 0 | 57 |
|  | intermediate | 20 | 0 | 25 | 45 |
|  | high | 0 | 36 | 34 | 70 |
| Total patients | | 59 | 54 | 59 | 172 |

**B**

| **Reclassification table events** | | | | | |
| --- | --- | --- | --- | --- | --- |
| **Model** |  | IMPACT + hsTnT | | | Total patients |
|  | **Risk group** | low | intermediate | high |  |
| IMPACT | low | 1 | 4 | 0 | 5 |
|  | intermediate | 0 | 0 | 11 | 11 |
|  | high | 0 | 4 | 20 | 24 |
| Total patients | | 1 | 8 | 31 | 40 |

**Legend:** Tables show reclassification of risk for 1-year mortality in patients undergoing HTX by a model using IMPACT combined with hsTnT compared to a model using IMPACT alone. Table A shows reclassification in 172 patients who did not experience 1-year mortality (non-events). The new model including hsTnT correctly downrated the risk of 56 patients (true negative) while falsely uprated the risk of 43 patients (false positive). Table B shows reclassification in 40 patients who died within the first year after HTX (events). The new model including hsTnT correctly uprated the risk of 15 patients (true positive) while falsely downrated the risk of 4 patients (false negative).
